# Having a better home range does not reduce the cost of reproduction in Soay sheep

**DOI:** 10.1111/jeb.14083

**Published:** 2022-09-05

**Authors:** Charlotte E. Regan, Josephine M. Pemberton, Jill G. Pilkington, Per T. Smiseth

**Affiliations:** ^1^ Institute for Evolutionary Biology, University of Edinburgh Edinburgh UK; ^2^ Department of Zoology Edward Grey Institute, University of Oxford Oxford UK

**Keywords:** costs of reproduction, home range quality, resource acquisition, Soay sheep

## Abstract

A cost of reproduction may not be observable in the presence of environmental or individual heterogeneity because they affect the resources available to individuals. Individual space use is critical in determining both the resources available to individuals and the exposure to factors that mediate the value of these resources (e.g. competition and parasitism). Despite this, there has, to our knowledge, been little research to understand how between‐individual differences in resource acquisition, caused by variation in space use, interact with environmental variation occurring at the population scale to influence estimates of the cost of reproduction in natural populations. We used long‐term data from the St. Kilda Soay sheep population to understand how differences in age, relative home range quality, and average adult body mass, interacted with annual variation in population density and winter North Atlantic Oscillation index to influence over‐winter survival and reproduction in the subsequent year, for females that had invested into reproduction to varying degrees. Our results suggest that Soay sheep females experience costs both in terms of future survival and future reproduction. However, we found little evidence that estimated costs of reproduction vary depending on relative home range quality. There are several possible causes for the lack of a relationship between relative home range quality and our estimate of the costs experienced by females. These include the potential for a correlation between relative home range quality and reproductive allocation to mask a relationship between home range quality and reproductive costs, as well as the potential for the benefit of higher quality home ranges being offset by higher densities. Nevertheless, our results raise questions regarding the presence or context‐dependence of relationships between resource access and the estimated cost of reproduction.

## Graphical abstract

Female St. Kilda Soay sheep anad lamb.

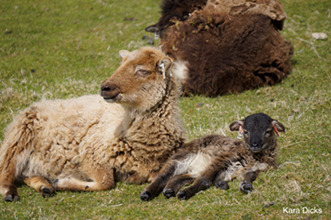

## 1 INTRODUCTION

Organisms are faced with choices over how to use the limited resources available to them, with individuals expected to partition these resources for investment in growth, reproduction, and survival such that their expected fitness is maximized (Stearns, 1989; Williams, 1966). This is expected to give rise to trade‐offs, where increased allocation in one trait necessitates reduced allocation in another (Stearns, 1989). For example, the increased allocation of resources to a reproductive event is expected to result in reduced survival and/or future reproduction, a trade‐off referred to as the ‘cost of reproduction’ (Bell, 1980). The cost of reproduction has been the focus of extensive study due to its potential role in shaping population dynamics (Hutchings, 1999; Jacquemyn et al., 2010) and possible consequences for the evolution of reproductive tactics (Bell, 1980). Such work has illustrated a significant cost of reproduction across a wide range of vertebrates (Bleu et al., 2016; Koivula et al., 2003; Moyes et al., 2006; Nager et al., 2001; Tavecchia et al., 2001) and invertebrates (Creighton et al., 2009; Kotiaho & Simmons, 2003; Papadopoulos et al., 2010; Scharf et al., 2013). However, negative correlations between current reproduction and future survival and/or reproduction are not always found (Hare & Murie, 1992; Pettifor et al., 1988; Santos & Nakagawa, 2012). In fact, positive correlations between allocation to current reproduction and future performance have been reported in multiple species, including deer mice (*Peromyscus maniculatus*) (Millar et al., 1992) and willow tits (*Poecile montanus*) (Orell et al., 1996). Such unexpected results have raised questions about the factors that may mask life‐history trade‐offs, or generate differences in the costs estimated in species from different taxonomic groups (Hamel, Gaillard et al., 2010).

The lack of consistent evidence for a cost of reproduction may be explained by both environmental and individual heterogeneity due to their possible influence on the amount of resources acquired by individuals. The costs associated with increased allocation to current reproduction may be more pronounced when conditions are particularly harsh, for example, at high density (Clutton‐Brock et al., 1996; Festa‐Bianchet et al., 1998; Hamel, Côté et al., 2010) or when winter conditions are severe (Barbraud & Weimerskirch, 2005; Tavecchia et al., 2005). In contrast, if resources are plentiful, individuals may be able to compensate for the cost of reproduction by increasing their intake (Bonnet et al., 2002; Ruckstuhl & Festa‐Bianchet, 2010). Similarly, between‐individual differences in resource acquisition might explain why studies often fail to detect a cost of reproduction, or may even lead to counter‐intuitive positive correlations between reproductive allocation and survival and/or future reproduction (Festa‐Bianchet et al., 2019), something that was first proposed by van Noordwijk and de Jong (1986). There is good evidence that life‐history trade‐offs depend on individual age (Tavecchia et al., 2001; Descamps et al., 2009; Hamel, Côté et al., 2010), body mass (Festa‐Bianchet et al., 1998), condition (Cichoń et al., 2008), or quality (Hamel, Côté et al. 2009; Hamel, Gaillard et al. 2009; Hamel, Côté et al., 2010). However, despite the importance of access to resources as a source of individual heterogeneity, to our knowledge, little is known about how differences in individual space use may mediate the estimated cost of reproduction. This is even though space use is likely to be critical in determining the resources accessible to individuals where there is fine‐scale spatial variability in the environment. Furthermore, where there is variation in the quantity or quality of resources, there is also likely to be variation in the density of con‐ and hetero‐specifics. Therefore, space use may also dictate the exposure of individuals to competition and parasitism, both of which may offset any gains in terms of resource access. Studies attempting to understand how resource acquisition may affect estimates of reproductive costs have largely exploited variability in resource availability between years or populations, often using proxies of resource availability, such as population density (e.g. Hamel, Côté et al., 2010; Moyes et al., 2011; Toni et al., 2020), even though such proxies will often be associated with variability in factors such as competition, parasite or disease exposure, and predation risk. Thus, despite the recognition that differences in individual resource acquisition can mask the cost of reproduction, we know little about if or how differences in individual space use translate into variation in the estimated cost of reproduction.

We aimed to understand (i) whether accounting for differences in resource access due to variation in home range quality affects estimates of the reproductive costs incurred by females, and (ii) if we were only able to detect costs of reproduction in certain individuals or under certain environmental conditions. We focus on females only given that we cannot accurately quantify resource allocation by males into the rut, that greater allocation of resources during this time does not necessarily translate into greater paternity, and that male space use varies markedly before and during the rut. We used data from the long‐term individual‐based study of St. Kilda Soay sheep. Soay sheep are believed to descend from some of the first domesticated sheep in Europe (Campbell, 1974; Chessa et al., 2009) and to have occupied the St. Kilda archipelago for 3000–4000 years (Clutton‐Brock et al., 2004a). The population residing in the Village Bay area on the Island of Hirta has been studied since 1985, with individuals followed from birth until death via frequent surveys. Most prime‐aged females reproduce each year and there is evidence that female survival is reduced following reproduction, with the cost varying according to density, weather, and age (Clutton‐Brock et al., 1996; Tavecchia et al., 2005). Furthermore, there is substantial variation in forage quality and quantity across the Village Bay area, partially due to previous patterns of human cultivation (permanent residents evacuated the island in 1930). Though the sheep will use a variety of grassland and heathland communities, *Holcus‐Agrostis* (HA) grassland is the most productive plant community on the island (Crawley et al., 2004), being selected for even when the sheep are at high density (Jones et al., 2006). Prior research has shown that variation in grazing quality is a likely driver of spatial variation in survival, recruitment, and dispersal (Coulson et al., 1999). Similarly, the quality of a female's home range is associated with her lifetime reproductive success, but this does not seem to be driven by benefits in terms of lamb growth or survival (Regan et al., 2016, 2017). Thus, we predicted that (i) accounting for relative home range quality (the relative proportion of HA grassland in the home range) would be associated with increased estimates of the cost of reproduction, and that (ii) relative home range quality would alter a female's ability to cope with the demands associated with reproduction, and therefore, that the estimated cost of reproduction may depend on a female's relative home range quality.

## 2 MATERIALS AND METHODS

### 2.1 Survival and reproduction data

This study uses data for all females born in or after 1985, that were alive in the April following birth (the earliest point at which a female can reproduce), and that were known or believed to be dead by the spring of 2020 (see below). For each year of an individual's life, we classified their reproductive status as follows. First, we assigned individuals as having bred or not bred (as in previous studies, e.g. Tavecchia et al., 2005), based on whether they were seen with a lamb during the lambing season or were confirmed to have had a lamb using genetic data. Second, to capture the potential costs of lactation, we separated individuals that bred into two groups based on whether they did or did not wean their singleton lamb, or in the case of individuals that gave birth to twins, whether they did or did not wean at least one of their lambs (with weaning denoted by survival of lambs to their first August when they are 4 months old). Third, we also split those individuals that weaned at least one lamb into two groups based on whether they gave birth to a singleton or twins to better capture the potential costs of rearing twins.

To examine potential survival costs of reproduction, we determined survival over the winter when mortality is greatest, by recording whether females survived until May the year after a potential reproductive bout (Clutton‐Brock et al., 2004b). Frequent mortality surveys make it possible to determine the month in which an individual most likely died. Where possible, we used this information to determine survival over a given winter. However, it can be difficult to assign a month of death if individuals died between trips to the island. In such cases, we used any information on reproductive events and re‐sightings during censuses to determine whether an individual had survived to May of each year of life or not. Where an individual had been recorded as having died in a particular year, but we lacked information on a month of death and had no subsequent reproductive or census data, we classified them as having died. This is because most mortality in this population occurs in late winter (i.e. prior to May) and re‐sighting probability is close to 100%. To study costs in terms of reduced probability of reproducing in the subsequent spring, we classified whether females had reproduced in each year of life based on lambing observations and genetically assigned maternities in the case of lambs that died before being observed with their mother.

### 2.2 Quantifying individual and environmental variability

**FIGURE 1:**
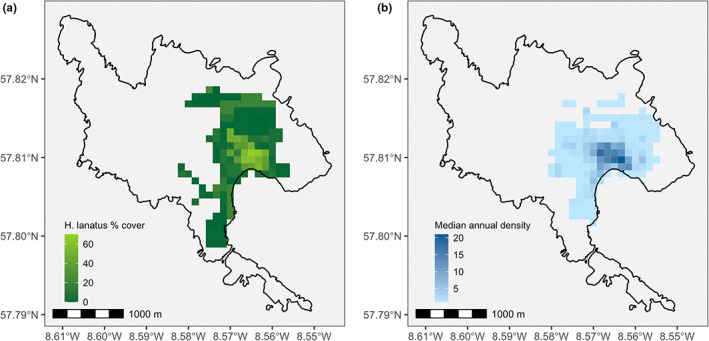
There is considerable spatial variability across Village Bay both in terms of the percentage cover of *Holcus lanatus* (a) and population density (b). Pixels corresponding to 1‐hectare sampling areas for botanical surveys and sheep censuses. We show the whole island of Hirta for scale, but the long‐term study of Soay sheep covers the Village Bay area in the southeast of the island

To capture variation in resource access due to differences in individual space use, we estimated each individual's relative home range quality in the year of reproduction as the relative mean percentage cover of *Holcus lanatus*, which is a key component of HA grassland (Figure 1). Female Soay sheep are strongly philopatric, and the proportion of HA grassland in home ranges can range from as little as 10% to as much as 62% (Regan et al., 2017). We estimated annual home ranges using census observations from the three census periods in each year (April/May, July/August, and October/November) using kernel density estimation methods in ‘adehabitatHR’ (Calenge, 2006). We estimated annual home range quality because although female home range quality remains relatively consistent over time, there might be small changes in quality over an individual's lifetime (mean change in HA cover = 7.89%, range = 0.01–25.63). We restricted our analyses to cases where individuals were observed at least 16 times during the year because incremental area analysis indicated that this is the number of observations needed to get an asymptote in core home range area (70% isopleth). Individuals can have up to 30 census observations per year. However, this approach led to the exclusion of some individuals in some years, as 125 (of 964) females did not have the necessary number of observations for every year of their life.

To prevent individuals from having multiple observations with the same coordinates which can make home range estimation problematic (Tufto et al., 1996), we added a random number between −20 and 20 (representing 20 m) to X and Y coordinates of each observation. We used the reference bandwidth (*h*_ref_) rather than the *ad hoc* bandwidth recommended by Kie (2013) when estimating home ranges because we have previously found that both methods produce comparable home range quality estimates (Regan et al., 2017). Between 2008 and 2012, 160 hectares of Village Bay were surveyed, and all vascular plants present in each hectare identified. At the same time, percentage cover of each species (to the nearest 5%) was scored by eye. For each annual home range, we identified the hectares contained within the 70% isopleth, and calculated the mean *H. lanatus* cover across the constituent hectares. We used the proportion of the hectare contained within the home range as a weight to ensure that hectares covering a greater proportion of the home range contributed more to the home range quality measure. Once we had annual estimates of home range quality (i.e. *H. lanatus* cover) for each individual, we year‐centred these measures so that each individual's annual home range quality is expressed relative to the average home range quality in that year.

We also included individual age at reproduction and individual mean residual body mass over their lifetime to capture individual heterogeneity that may also lead to variation in the costs of reproduction experienced by individuals. We classified individuals as yearlings (i.e. 1 year old), prime‐aged adults (2–7 years old), or old adults (>7 years old). We estimated an individual's lifetime mean residual body mass by taking August mass measurements for each individual in each year they were weighed (note that not all individuals are captured every year), extracting the residuals from a linear model with body mass as the response variable and foreleg length and age (linear and quadratic terms) as explanatory variables, and averaging these for each individual.

We included variables describing population density and winter weather in our models to understand how the inclusion of between‐year environmental variation affected our estimates of the costs of reproduction and whether there was any evidence that costs were more pronounced when conditions were poor. To capture variation in population density, we used the number of adults (> 1 year old) in the Village Bay area recorded across the 10 August censuses in the year of the reproductive bout. Owing to sheep being marked with unique ear tags, double counting of individuals is very unlikely. In addition, because we use 10 censuses to derive summer density estimates, it is also unlikely that individuals would have been missed entirely. To characterize variation in the severity of winter weather conditions, we used the North Atlantic Oscillation index (NAO), which captures the effect of winter weather on ungulate survival and reproduction (Coulson et al., 2001; Catchpole et al., 2004; Pioz et al., 2008, but see Kjellander et al., 2006; Martínez‐Jauregui et al., 2009). The NAO is calculated as the difference between the normalized sea‐level pressure at weather stations in Ponta Delgada (Portugal) and Reykjavik (Iceland), and winter NAO (average over December–March) provides a measure of winter weather in Western Europe (Hurrell, 1995). High NAO values correspond to mild, wet, and stormy winters that are associated with reduced over‐winter survival in St. Kilda Soay sheep (Coulson et al., 2001). We obtained NAO values for the period of 1985–2020 from https://crudata.uea.ac.uk/cru/data/nao and averaged values from December to March of each year to obtain a measure of weather severity in the winter following each reproductive bout.

### 2.3 Statistical analysis

We tested for effects of our three measures of reproductive allocation on the probability of over‐winter survival, probability of reproduction in the subsequent year, and the probability of twinning in three separate model sets. In each case, our models followed the same structure but used a single measure of reproductive allocation (i.e. bred vs. did not breed; weaned lamb vs. did not wean; weaned twin vs. weaned singleton). However, it is important to note that in analyses considering individuals that weaned a singleton versus those that weaned twin(s), we excluded yearling females as they never bear twins.

To understand how estimates of the cost of reproduction are affected by accounting for variation in individual resource access and environmental conditions, we first used three models to understand how accounting for individual variation in long‐term body mass or relative home range quality, and between‐year variability in density and winter weather affected our estimates of reproductive costs. The first model contained only the measure of reproductive status and female age (three‐level factor) (base model/model 1 in Tables S1–S7); the second included these terms as well as an individual's mean residual body mass and their relative home range quality (model 2 in Tables S1–S7); the third included all prior terms as well as population density and NAO (model 3 in Tables S1–S7). A comparison of these models thus allows us to assess support for the effects of between‐individual and inter‐annual differences on survival and reproductive probability as well as how the inclusion of these variables alters our estimates of the cost of reproduction.

To understand whether estimated costs of reproduction varied according to individual resource acquisition and annual environmental conditions, we used an additional set of models to understand how our measures of individual resource acquisition and annual environmental variability predicted the magnitude of the estimated reproductive costs experienced by individuals. We considered first‐order interactions between reproductive status and each measure of individual resource access (age, mean residual body mass, relative home range quality) and annual environmental conditions (population density and NAO) to understand how reproductive costs were mediated by variation in resource access and environmental conditions (models 4 – 8 in Tables S1–S7). We also considered second‐order interactions between reproductive status, age, and each of the other four terms (mean residual body mass, relative home range quality, density, and NAO) to understand whether the degree to which estimated costs were influenced by variation in resource access or annual conditions varied between individuals of different ages (models 9 – 14 in Tables S1–S7). We then used second‐order interactions between reproductive status, mean residual weight/relative home range quality, and both density and NAO to understand whether any influence of resource access on the estimated magnitude of reproductive costs varied according to broader environmental conditions following breeding (models 15 and 16 in Tables S1–S7). Finally, we considered the second‐order interaction between reproductive status, population density, and NAO to understand whether the estimated costs of reproducing at high density were affected by winter weather conditions, as previously shown by Tavecchia et al. (2005) (model 17 in Tables S1–S7). See Tables S1–S7 for all model structures.

In each case, we used generalized linear mixed models with a binomial distribution and logit link function and included the year of breeding and individual identity as random effects. All covariates were scaled to a mean of zero and standard deviation of 1 prior to analysis to enable direct comparisons, and we used Akaike's Information Criterion corrected for small sample size (AIC_c_) to compare the relative support for models in each model set. All data manipulation and analysis were carried out in R version 4.0.3 using sf (version 1.0–2), adehabitatHR (version 0.4.18), dplyr (version 1.0.4), and lme4 (version 1.1–25) packages.

## 3 RESULTS

### 3.1 Subsequent survival

Breeding females were 3% less likely to survive over the next winter than non‐breeding females (Model 1 – breeders: 0.95 ± 0.01, non‐breeders: 0.98 ± 0.01 [est ± SE]), suggesting a small cost of reproduction in terms of reduced overwinter survival. This difference increased slightly to 4% when we considered females that had invested into lactation versus those that had not (Model 1 – weaned: 0.95 ± 0.01, did not wean: 0.99 ± 0.004 [est ± SE]). Weaning twins was not more costly than weaning a singleton lamb (Model 1 – weaned twins: 0.95 ± 0.02, weaned singleton: 0.96 ± 0.01 [est ± SE]). Including variables describing individual heterogeneity (relative home range quality and mean residual body mass) and variation in annual environmental conditions improved model fit (bred vs did not breed: ΔAIC_c_ = −52.54, weaned vs did not wean: ΔAIC_c_ = −32.53, twins vs singleton: ΔAIC_c_ = −14.62; Tables S2A–S4A), but did not alter the magnitude of the estimated effect of reproductive status on survival probability (Table 1).

**TABLE 1:** Estimates and standard errors from survival models where resource acquisition and annual environmental variability were and were not accounted for as fixed effects. Statistically significant effects (*p* < 0.05) are given in bold

	Base model		Including individual and environmental heterogeneity	
	Est	SE	Est	SE
Bred vs did not breed				
Intercept	**4.16**	**0.33**	**3.92**	**0.23**
Reproductive status (bred)	**−0.68**	**0.19**	**−0.54**	**0.19**
Maternal age (old)	**−1.92**	**0.13**	**−2.01**	**0.13**
Maternal age (yearling)	**−0.65**	**0.20**	**−0.56**	**0.20**
Mean residual body mass	–	–	**0.43**	**0.06**
Relative home range quality	–	–	0.10	0.06
Density	–	–	−0.55	0.31
NAO	–	–	0.16	0.33
Weaned vs did not wean				
Intercept	**4.90**	**0.38**	**4.82**	**0.38**
Reproductive status (weaned)	**−1.41**	**0.25**	**−1.48**	**0.26**
Maternal age (old)	**−2.04**	**0.14**	**−2.08**	**0.14**
Maternal age (yearling)	**−1.36**	**0.27**	**−1.40**	**0.28**
Mean residual body mass	–	–	**0.37**	**0.07**
Relative home range quality	–	–	0.11	0.07
Density	–	–	**−0.75**	**0.32**
NAO	–	–	0.02	0.33
Twin vs singleton				
Intercept	**3.59**	**0.32**	**3.45**	**0.31**
Reproductive status (twins)	−0.24	0.19	−0.31	0.19
Maternal age (old)	**−2.04**	**0.15**	**−2.06**	**0.15**
Mean residual body mass	–	–	**0.27**	**0.07**
Relative home range quality	–	–	0.12	0.07
Density	–	–	**−0.66**	**0.37**
NAO	–	–	0.14	0.38

**FIGURE 2:**
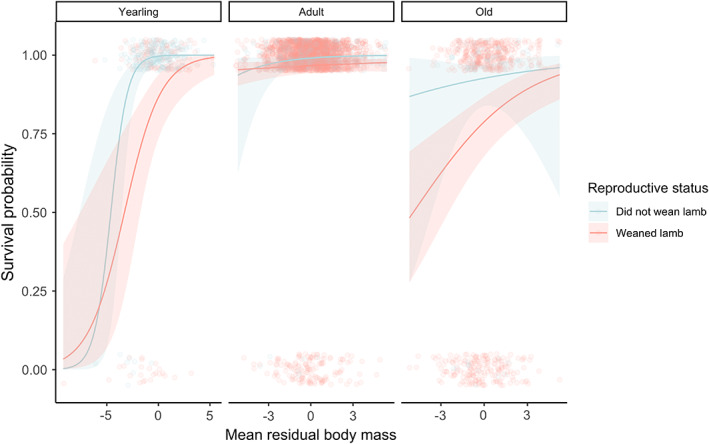
Mean age‐corrected residual body mass was a key predictor of female over winter survival probability, particularly for yearling females (1 year old) and old (8+ years old) breeding females. Solid lines correspond to the fitted relationships from mixed models and shaded areas to 95% confidence intervals

When comparing the set of 15 models, we found that for all reproductive status metrics, the best‐fit model included the interaction between reproductive status, maternal age, and mean residual body mass (Model 9; Tables S2B–S4B); however, when comparing females that reared twins with those that reared singletons, two other models had comparable AIC_c_ (Table S4B). Best‐fit models indicated that heavier females were more likely to survive the winter (bred vs did not breed: *β* = 0.43, SE = 0.06, *p* < 0.001, weaned vs did not wean: *β* = 0.36, SE = 0.07, *p* < 0.001), and that the benefits of being relatively heavy were particularly pronounced for yearlings (regardless of reproductive status) and old breeding females (Figure 2). We found little evidence to suggest that estimated survival costs of reproduction in this population varied according to the quality of an individual's home range, given the lack of support for models including an interaction between relative home range quality and reproductive status (bred vs did not breed: ΔAIC_c_ = +28.28, est = −0.11, SE = 0.16, *p* = 0.52; weaned vs did not wean: ΔAIC_c_ = +17.52, est = 0.13, SE = 0.23, *p* = 0.59; twin vs singleton: ΔAIC_c_ = +2.85, est = 0.005, SE = 0.21, *p* = 0.98, Tables S2B–S4B).

### 3.2 Subsequent reproduction

When considering the cost of reproduction in terms of reduced reproduction in the subsequent year, our findings were somewhat dependent on the measure of reproductive status used. When we compared females that bred (irrespective of the degree of resource allocation) with those that had not bred, we found that non‐reproductive females were actually less likely to reproduce in the subsequent year, regardless of whether we included information on individual resource acquisition or annual environmental conditions (Models 1–3 in Tables S1–S7; Table 2). However, females that had weaned a lamb, and therefore invested into lactation, were 5% less likely to reproduce in the following spring compared to females that had not weaned their lamb (weaned: 0.85 ± 0.03, did not wean: 0.90 ± 0.02 [est ± SE]). This effect was particularly pronounced for yearling females, where individuals that successfully weaned their lamb were 24% less likely to reproduce in the subsequent year compared to those that did not raise their lamb to weaning (weaned: 0.63 ± 0.08, did not wean: 0.87 ± 0.04 [est ± SE]). Females that weaned one or both of their twins were 5% less likely to reproduce in the subsequent year than those that weaned only a singleton lamb (weaned twin(s): 0.81 ± 0.04, weaned singleton: 0.86 ± 0.03 [est ± SE]), suggesting a cost of rearing twins. However, this cost was only statistically significant when accounting for terms describing variation in individual resource acquisition and annual environmental conditions (Model 3; Table 2).

**TABLE 2:** Estimates and standard errors from reproductive probability models where resource acquisition and annual environmental variability were and were not accounted for as fixed effects. Statistically significant effects (*p* < 0.05) are given in bold

	Base model		Including individual and environmental heterogeneity	
	Est	SE	Est	SE
Bred vs did not breed				
Intercept	**1.34**	**0.27**	**1.25**	**0.28**
Reproductive status (bred)	**0.69**	**0.15**	**0.68**	**0.15**
Maternal age (old)	**−2.15**	**0.14**	**−2.18**	**0.14**
Maternal age (yearling)	−0.11	0.15	−0.10	0.15
Mean residual body mass	–	–	**0.22**	**0.06**
Relative home range quality	–	–	0.03	0.06
Density	–	–	−0.33	0.26
NAO	–	–	0.01	0.29
Weaned vs did not wean				
Intercept	**2.62**	**0.28**	**2.49**	**0.28**
Reproductive status (weaned)	**−0.45**	**0.16**	**−0.46**	**0.16**
Maternal age (old)	**−1.91**	**0.14**	**−1.92**	**0.14**
Maternal age (yearling)	**−0.92**	**0.20**	**−0.94**	**0.20**
Mean residual body mass	–	–	**0.26**	**0.06**
Relative home range quality	–	–	0.08	0.05
Density	–	–	**−0.53**	**0.26**
NAO	–	–	−0.05	0.29
Twin vs singleton				
Intercept	**2.28**	**0.26**	**2.12**	**0.25**
Reproductive status (twins)	−0.30	0.16	**−0.33**	**0.16**
Maternal age (old)	**−1.71**	**0.15**	**−1.69**	**0.15**
Mean residual body mass	–	–	**0.20**	**0.06**
Relative home range quality	–	–	**0.12**	**0.06**
Density	–	–	**−0.68**	**0.30**
NAO	–	–	−0.02	0.31

**FIGURE 3:**
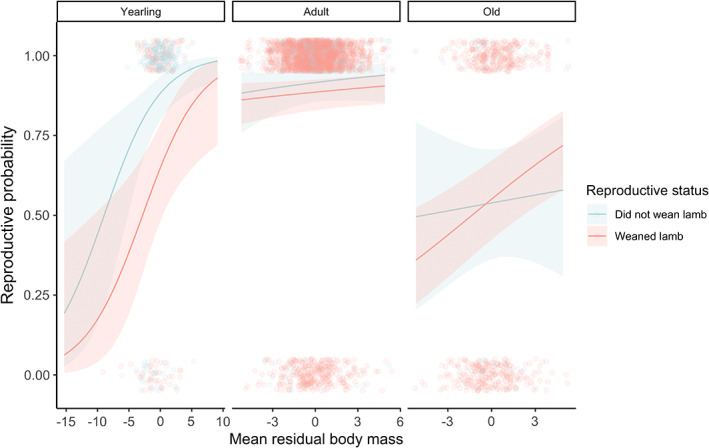
Mean age‐corrected residual body mass was a key predictor of a female's likelihood of reproduction in the subsequent spring, particularly for those that had bred and weaned a lamb in the current breeding season. Solid lines correspond to the fitted relationships from mixed models and shaded areas to 95% confidence intervals

**FIGURE 4:**
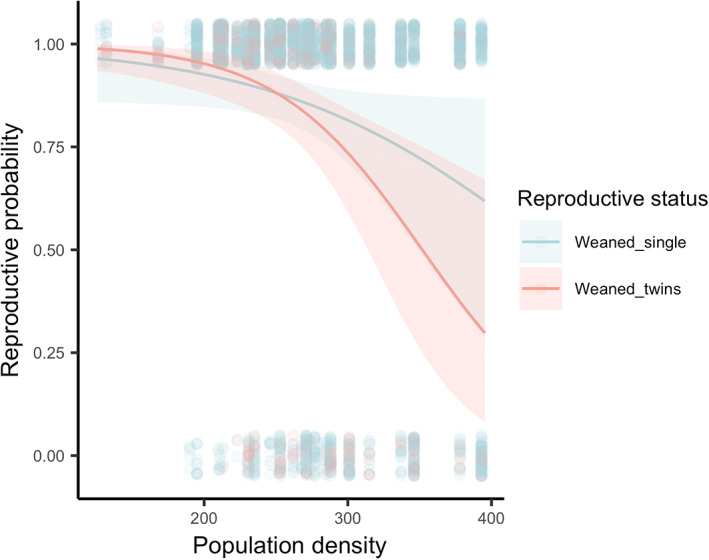
A female's subsequent reproductive probability was only reduced at high density when they had invested into reproduction in the current year. Solid lines correspond to the fitted relationships from mixed models, and shaded areas to 95% confidence intervals.

When comparing females that bred versus those that did not breed, and those that weaned their lamb versus those that did not, the best fit model included an interaction between reproductive status, maternal age, and mean residual body mass (Model 9; Tables S5B–S7B). Reproductive females across all ages were more likely to reproduce the following year if they were heavier (Figure 3), whilst there was no clear relationship between mean residual body mass and reproductive probability for non‐breeders, except for yearlings, where being heavier was beneficial regardless of their reproductive status (Figure 3). When comparing females that reared twins versus those with singletons, the best‐fit model included an interaction between reproductive status and density (Model 7; though there were two other models within two AIC_c_ units of this model). Reproductive probability was reduced at high density for both females that had twinned and those that reared singletons, but this reduction was stronger for those providing care to two lambs (Figure 4).

As for models of subsequent survival probability, models including interactions between reproductive status and relative home range quality were not competitive according to AIC_c_ (Tables S5B–S7B). Therefore, we have little evidence to suggest that estimated reproductive costs in this population were affected by the quality of a female's home range.

## 4 DISCUSSION

As in previous work in this system, we found that female Soay sheep incur a cost of reproduction in terms of reduced future survival (Clutton‐Brock et al., 1996; Leivesley et al., 2019; Tavecchia et al., 2005). We also found evidence that females experienced a cost in terms of a reduced probability to reproduce in the subsequent year. We found little evidence that the estimated cost of reproduction in this system depended on the quality of the home range occupied by an individual, although it did vary depending on both individual age and mean residual body mass, and to some degree on annual density. The lack of a relationship between relative home range quality and the cost of reproduction raises questions regarding the presence or context‐dependence of relationships between access to resources and the estimated magnitude of life‐history trade‐offs such as the cost of reproduction. Below, we discuss potential reasons for the lack of a relationship in our case and potential avenues for further research.

Our finding, that relative home range quality seemingly has little influence on estimates of the survival or reproductive costs of reproduction for female Soay sheep indicates that the relationship between individual resource access and life‐history trade‐offs may be more complex than might have previously been assumed. In line with studies at the population level (Toni et al., 2020; Török et al., 2004), we expected that individuals with greater access to resources to be less likely to experience a cost as a result of reproduction. It is unlikely that the lack of an effect of relative home range quality found here is due to a shortage of data or an inappropriate characterization of the quality of resources available to females given the size of the dataset (4298 reproductive years from 839 females), quality of survival and reproductive data, and the employment of a relative home range quality metric previously shown to correlate with survival and reproductive success in this system (Froy et al., 2018; Regan et al., 2016). Instead, we suggest that the lack of a relationship between relative home range quality and the estimated cost of reproduction might provide valuable insights into when we may or may not expect variability in access to resources to translate into variability in the estimated costs experienced by individuals, and thereby mask trade‐offs at the population level.

One potential reason for the lack of a relationship between relative home range quality and estimated costs of reproduction in Soay sheep may lie in the way that Soay sheep females finance reproduction. Species are often placed along a spectrum from capital to income breeders, with capital breeders, such as Soay sheep, relying heavily on stored resources to finance reproduction and income breeders relying on current resources to sustain reproduction (Stearns, 1992). A consequence of this is that capital breeders tend to show a strong correlation between body reserves and fecundity (Boyd, 2000). Therefore, if greater access to high‐quality forage facilitates increased allocation in reproduction, we might expect a relationship between relative home range quality and fecundity. This is precisely what we found previously for Soay sheep females (Regan et al., 2016). Essentially, in this system, a lack of a relationship between relative home range quality and estimated costs of reproduction may be driven by differences in the likelihood of such females reproducing or their relative allocation into reproduction. Thus, we might expect territory or relative home range quality to be more important in moderating the costs of reproduction in income breeders that are more reliant on concurrent intake of resources to finance their allocation to reproduction (Boyd, 2000). Lack of research currently makes it difficult to draw conclusions on this point, thus highlighting the need for more research exploring the links between individual level resource availability and estimated costs of reproduction.

Another possible explanation for our results is the potential for resource quality to be offset by other factors varying spatially across Village Bay. Habitat selection is density‐dependant (McLoughlin et al., 2010; van Beest et al., 2014), with variation in population density modifying the relative value of low‐ and high‐quality habitats by altering the level of exploitation by conspecifics (Avgar et al., 2020). Such a process may have been a factor in our case given that Soay sheep do not conform to the ideal free distribution, with HA grassland being used by a greater proportion of the population than expected based on its availability (Jones et al., 2006, Figure 1). Where resource availability or quality and local population density are correlated, we may also see correlations between resource availability/quality and other factors that may modify the value of resources to individuals. For example, we may expect population density to correlate with parasite exposure (Body et al., 2011; Hayward et al., 2014; Wilson et al., 2004), potentially leading to higher parasite burden in areas of high quality. Work is revealing the role that parasitism may play in mediating trade‐offs such as the cost of reproduction (Albery et al., 2020; Leivesley et al., 2019), further highlighting the complexity associated with quantifying between‐individual variation in resource acquisition and the likely need to model various factors altering individual energy balances.

Overall, there is still substantial scope for further studies regarding if/how the resources available to an individual shape life‐history trade‐offs. For example, it is possible that the benefits of having a high‐quality home range are only apparent when considering costs manifested over longer time scales. Although this was beyond the scope of this piece of work, it may be possible to understand how variation in resource access mediates estimates of long‐term costs of reproduction in this and other systems, though such work will require careful consideration of how to quantify resource access over multiple years or the lifetime, particularly given that age‐related changes in space use may be expected (Froy et al., 2018). We suggest that understanding how resource access/use affects the presence/magnitude of trade‐offs will require detailed information on the value of different resources as well as how their value is affected by other factors, such as competition and exposure to parasites. It is also important to consider that individuals are not static in the resources they use, with habitat/resource use often varying temporally (Tsalyuk et al., 2019; Zweifel‐Schielly et al., 2009). Thus, individuals may alter their specific resource selection depending on their allocation to reproduction (Smith et al., 2018; Viejou et al., 2018). To our knowledge, little work has explored how such behaviour may mask life‐history trade‐offs; however, work has begun to highlight the likely link between behaviour and resource acquisition, and thus between behavioural variation and life‐history trade‐offs (Laskowski et al., 2021). Therefore, there is still much to learn regarding the ways that spatial variability in resource availability and individual behaviour interact to shape‐observed patterns in life‐history trade‐offs.

## AUTHOR CONTRIBUTIONS

C.E.R. conceived the study with inputs from P.T.S. and J.M.P. Data collection was overseen by J.G.P. C.E.R. performed the analysis and drafted the manuscript with input from P.T.S. and J.M.P.

## CONFLICT OF INTEREST STATEMENT

The authors declare no competing interests.

### OPEN RESEARCH BADGES

Is the author interested in applying for an Open Research Badge?:Yes

### PEER REVIEW

RESPONSE TO PEER REVIEW TRANSPARENCY OPTION (reviewer reports, author responses, and decision letter linked from Publons): Agree

## Supporting information


Tables S1–S7

## Data Availability

All data necessary to replicate the results have been archived on Figshare (https://doi.org/10.6084/m9.figshare.20059160.v1).
