# Supplementary material for: Having a better home range does not reduce the cost of reproduction in Soay sheep
Source: J Evol Biol. 2022 Sep 5;35(10):1352–62. doi: 10.1111/jeb.14083 (PMC9826142; doi:10.1111/jeb.14083)
Supplement: Supplementary file 1 — Tables S1–S7 [file JEB-35-1352-s001.docx]

**SUPPORTING INFORMATION**

**Model structures**

**Table 1.** Fixed and random effect structures for all models described in the analyses. Model structures were the same for both survival and reproductive analyses.

| Model | Fixed effects | Interactions | Random effects |
| --- | --- | --- | --- |
| 1 | Reproductive status + maternal age | - | Year + ID |
| 2 | Reproductive status + maternal age + body mass + home range quality | - | Year + ID |
| 3 | Reproductive status + maternal age + body mass + home range quality + density + NAO | - | Year + ID |
| 4 | Reproductive status + maternal age + body mass + home range quality + density + NAO | Reproductive status * maternal age | Year + ID |
| 5 | Reproductive status + maternal age + body mass + home range quality + density + NAO | Reproductive status * body mass | Year + ID |
| 6 | Reproductive status + maternal age + body mass + home range quality + density + NAO | Reproductive status * home range quality | Year + ID |
| 7 | Reproductive status + maternal age + body mass + home range quality + density + NAO | Reproductive status * density | Year + ID |
| 8 | Reproductive status + maternal age + body mass + home range quality + density + NAO | Reproductive status * NAO | Year + ID |
| 9 | Reproductive status + maternal age + body mass + home range quality + density + NAO | Reproductive status * maternal age * body mass | Year + ID |
| 10 | Reproductive status + maternal age + body mass + home range quality + density + NAO | Reproductive status * maternal age * home range quality | Year + ID |
| 11 | Reproductive status + maternal age + body mass + home range quality + density + NAO | Reproductive status * maternal age * density | Year + ID |
| 12 | Reproductive status + maternal age + body mass + home range quality + density + NAO | Reproductive status * maternal age * NAO | Year + ID |
| 13 | Reproductive status + maternal age + body mass + home range quality + density + NAO | Reproductive status * body mass * density | Year + ID |
| 14 | Reproductive status + maternal age + body mass + home range quality + density + NAO | Reproductive status * body mass * NAO | Year + ID |
| 15 | Reproductive status + maternal age + body mass + home range quality + density + NAO | Reproductive status * home range quality * density | Year + ID |
| 16 | Reproductive status + maternal age + body mass + home range quality + density + NAO | Reproductive status * home range quality * NAO | Year + ID |
| 17 | Reproductive status + maternal age + body mass + home range quality + density + NAO | Reproductive status * density * NAO | Year + ID |

**AIC_c_ values for survival models**

**Table 2A.** AICc comparisons for the three models used to understand how accounting for individual characteristics and environmental conditions altered estimates of over-winter survival when comparing females that bred versus those that did not.

| *Model* | *df* | *AICc* | *delta* | *weight* |
| --- | --- | --- | --- | --- |
| 3 | 10 | 2043.08 | 0.00 | 0.76 |
| 2 | 8 | 2045.38 | 2.30 | 0.24 |
| 1 | 6 | 2095.62 | 52.54 | 0.00 |

**Table 2B.** AICc comparisons for the 15 models used to understand whether the effect of breeding versus not breeding on subsequent survival probability varied depending on individual characteristics or environmental conditions

| *Model* | *df* | *AICc* | *delta* | *weight* |
| --- | --- | --- | --- | --- |
| 9 | 17 | 2016.39 | 0.00 | 1.00 |
| 5 | 11 | 2038.77 | 22.38 | 0.00 |
| 14 | 14 | 2040.76 | 24.36 | 0.00 |
| 13 | 14 | 2041.42 | 25.03 | 0.00 |
| 17 | 14 | 2042.66 | 26.26 | 0.00 |
| 7 | 11 | 2042.88 | 26.49 | 0.00 |
| 3 | 10 | 2043.08 | 26.68 | 0.00 |
| 6 | 11 | 2044.67 | 28.28 | 0.00 |
| 8 | 11 | 2044.94 | 28.55 | 0.00 |
| 15 | 14 | 2046.39 | 30.00 | 0.00 |
| 4 | 12 | 2047.05 | 30.66 | 0.00 |
| 11 | 17 | 2049.19 | 32.80 | 0.00 |
| 16 | 14 | 2050.03 | 33.64 | 0.00 |
| 12 | 17 | 2053.84 | 37.45 | 0.00 |
| 10 | 17 | 2055.32 | 38.93 | 0.00 |

**Table 3A** AICc comparisons for the three models used to understand how accounting for individual characteristics and environmental conditions altered estimates of over-winter survival when comparing females that weaned a lamb to those that did not.

| *Model* | *df* | *AICc* | *delta* | *weight* |
| --- | --- | --- | --- | --- |
| 3 | 10 | 1718.86 | 0.00 | 0.90 |
| 2 | 8 | 1723.17 | 4.31 | 0.10 |
| 1 | 6 | 1751.39 | 32.53 | 0.00 |

**Table 3B.** AICc comparisons for the 15 models used to understand whether the effect of weaning a lamb versus not weaning a lamb on subsequent survival probability varied depending on individual characteristics or environmental conditions

| *Model* | *df* | *AICc* | *delta* | *weight* |
| --- | --- | --- | --- | --- |
| 9 | 17 | 1703.07 | 0.00 | 1.00 |
| 5 | 11 | 1714.99 | 11.93 | 0.00 |
| 17 | 14 | 1718.77 | 15.70 | 0.00 |
| 3 | 10 | 1718.86 | 15.79 | 0.00 |
| 13 | 14 | 1719.33 | 16.26 | 0.00 |
| 4 | 12 | 1719.49 | 16.42 | 0.00 |
| 14 | 14 | 1719.83 | 16.76 | 0.00 |
| 8 | 11 | 1720.24 | 17.17 | 0.00 |
| 6 | 11 | 1720.59 | 17.52 | 0.00 |
| 7 | 11 | 1720.83 | 17.76 | 0.00 |
| 15 | 14 | 1724.56 | 21.49 | 0.00 |
| 16 | 14 | 1725.43 | 22.37 | 0.00 |
| 12 | 17 | 1725.87 | 22.80 | 0.00 |
| 11 | 17 | 1727.37 | 24.30 | 0.00 |
| 10 | 17 | 1728.39 | 25.32 | 0.00 |

**Table 4A.** AICc comparisons for the three models used to understand how accounting for individual characteristics and environmental conditions altered estimates of over-winter survival when comparing females that weaned a singleton lamb to those that weaned one or both twins.

| *Model* | *df* | *AICc* | *delta* | *weight* |
| --- | --- | --- | --- | --- |
| 3 | 10 | 1464.49 | 0.00 | 0.74 |
| 2 | 8 | 1466.62 | 2.14 | 0.26 |
| 1 | 6 | 1479.11 | 14.26 | 0.00 |

**Table 4B.** AICc comparisons for the 15 models used to understand whether the effect of weaning a singleton lamb versus twins on subsequent survival probability varied depending on individual characteristics or environmental conditions

| *Model* | *df* | *AICc* | *delta* | *weight* |
| --- | --- | --- | --- | --- |
| 9 | 13 | 1463.65 | 0.00 | 0.23 |
| 5 | 10 | 1463.99 | 0.34 | 0.20 |
| 3 | 9 | 1464.49 | 0.84 | 0.15 |
| 4 | 10 | 1466.15 | 2.50 | 0.07 |
| 8 | 10 | 1466.37 | 2.72 | 0.06 |
| 7 | 10 | 1466.46 | 2.82 | 0.06 |
| 6 | 10 | 1466.50 | 2.85 | 0.06 |
| 17 | 13 | 1467.38 | 3.74 | 0.04 |
| 13 | 13 | 1467.84 | 4.20 | 0.03 |
| 10 | 13 | 1467.85 | 4.20 | 0.03 |
| 14 | 13 | 1469.83 | 6.18 | 0.01 |
| 16 | 13 | 1470.67 | 7.02 | 0.01 |
| 15 | 13 | 1470.70 | 7.05 | 0.01 |
| 12 | 13 | 1471.49 | 7.85 | 0.00 |
| 11 | 13 | 1471.94 | 8.30 | 0.00 |

**AIC_c_ values for reproduction models**

**Table 5A.** AICc comparisons for the three models used to understand how accounting for individual characteristics and environmental conditions altered estimates of subsequent reproductive probability when comparing females that bred versus those that did not.

| *Model* | *df* | *AICc* | *delta* | *weight* |
| --- | --- | --- | --- | --- |
| 2 | 8 | 3642.20 | 0.00 | 0.70 |
| 3 | 10 | 3643.91 | 1.71 | 0.30 |
| 1 | 6 | 3651.57 | 9.36 | 0.01 |

**Table 5B.** AICc comparisons for the 15 models used to understand whether the effect of breeding versus not breeding on subsequent reproductive probability varied depending on individual characteristics or environmental conditions

| *Model* | *df* | *AICc* | *delta* | *weight* |
| --- | --- | --- | --- | --- |
| 9 | 17 | 3584.43 | 0.00 | 1.00 |
| 13 | 14 | 3627.02 | 42.59 | 0.00 |
| 10 | 17 | 3627.89 | 43.46 | 0.00 |
| 11 | 17 | 3629.67 | 45.24 | 0.00 |
| 15 | 14 | 3631.29 | 46.86 | 0.00 |
| 7 | 11 | 3632.39 | 47.96 | 0.00 |
| 17 | 14 | 3636.09 | 51.66 | 0.00 |
| 4 | 12 | 3636.58 | 52.15 | 0.00 |
| 14 | 14 | 3636.67 | 52.24 | 0.00 |
| 5 | 11 | 3638.76 | 54.33 | 0.00 |
| 12 | 17 | 3639.94 | 55.51 | 0.00 |
| 8 | 11 | 3641.23 | 56.80 | 0.00 |
| 6 | 11 | 3642.75 | 58.32 | 0.00 |
| 16 | 14 | 3642.87 | 58.44 | 0.00 |

**Table 6A.** AICc comparisons for the three models used to understand how accounting for individual characteristics and environmental conditions altered estimates of subsequent reproductive probability when comparing females that weaned their lamb versus those that did not

| *Model* | *df* | *AICc* | *delta* | *weight* |
| --- | --- | --- | --- | --- |
| 3 | 10 | 2812.37 | 0.00 | 0.65 |
| 2 | 8 | 2813.60 | 1.23 | 0.35 |
| 1 | 6 | 2833.90 | 21.53 | 0.00 |

**Table 6B.** AICc comparisons for the 15 models used to understand whether the effect of weaning a lamb versus not weaning a lamb on subsequent reproductive probability varied depending on individual characteristics or environmental conditions

| *Model* | *df* | *AICc* | *delta* | *weight* |
| --- | --- | --- | --- | --- |
| 9 | 17 | 2803.27 | 0.00 | 0.42 |
| 11 | 17 | 2803.86 | 0.59 | 0.31 |
| 4 | 12 | 2806.05 | 2.78 | 0.10 |
| 17 | 14 | 2808.06 | 4.79 | 0.04 |
| 15 | 14 | 2808.08 | 4.81 | 0.04 |
| 10 | 17 | 2808.26 | 4.99 | 0.03 |
| 12 | 17 | 2809.62 | 6.35 | 0.02 |
| 6 | 11 | 2810.76 | 7.49 | 0.01 |
| 7 | 11 | 2811.71 | 8.44 | 0.01 |
| 3 | 10 | 2812.37 | 9.10 | 0.00 |
| 8 | 11 | 2812.68 | 9.42 | 0.00 |
| 16 | 14 | 2813.49 | 10.22 | 0.00 |
| 5 | 11 | 2813.99 | 10.72 | 0.00 |
| 13 | 14 | 2816.53 | 13.26 | 0.00 |
| 14 | 14 | 2817.22 | 13.95 | 0.00 |

**Table 7A.** AICc comparisons for the three models used to understand how accounting for individual characteristics and environmental conditions altered estimates of subsequent reproductive probability when comparing females that weaned a singleton lamb versus those that weaned one or both twins

| *Model* | *df* | *AICc* | *delta* | *weight* |
| --- | --- | --- | --- | --- |
| 3 | 10 | 2240.59 | 0.00 | 0.83 |
| 2 | 8 | 2243.80 | 3.21 | 0.17 |
| 1 | 6 | 2256.36 | 15.77 | 0.00 |

**Table 7B.** AICc comparisons for the 15 models used to understand whether the effect of weaning one or both twins versus a singleton lamb on subsequent reproductive probability varied depending on individual characteristics or environmental conditions

| *Model* | *df* | *AICc* | *delta* | *weight* |
| --- | --- | --- | --- | --- |
| 7 | 10 | 2234.71 | 0.00 | 0.33 |
| 17 | 13 | 2234.81 | 0.11 | 0.31 |
| 16 | 13 | 2236.21 | 1.50 | 0.16 |
| 15 | 13 | 2238.28 | 3.58 | 0.06 |
| 8 | 10 | 2239.00 | 4.30 | 0.04 |
| 13 | 13 | 2240.01 | 5.30 | 0.02 |
| 11 | 13 | 2240.38 | 5.67 | 0.02 |
| 3 | 9 | 2240.59 | 5.89 | 0.02 |
| 5 | 10 | 2241.29 | 6.58 | 0.01 |
| 9 | 13 | 2241.92 | 7.22 | 0.01 |
| 4 | 10 | 2242.28 | 7.57 | 0.01 |
| 6 | 10 | 2242.35 | 7.64 | 0.01 |
| 14 | 13 | 2242.93 | 8.23 | 0.01 |
| 12 | 13 | 2244.50 | 9.79 | 0.00 |
| 10 | 13 | 2246.73 | 12.02 | 0.00 |
